# Exploring the Complex Interplay of Obesity, Allergic Diseases, and Sleep-Disordered Breathing in Children

**DOI:** 10.3390/children11050595

**Published:** 2024-05-15

**Authors:** Chiara Voltan, Francesca Concer, Luca Pecoraro, Angelo Pietrobelli, Giorgio Piacentini, Marco Zaffanello

**Affiliations:** Pediatric Clinic, Department of Surgery, Dentistry, Gynecology and Pediatrics, University of Verona, 37129 Verona, Italyangelo.pietrobelli@univr.it (A.P.);

**Keywords:** allergy, children, Down syndrome, inflammation, obesity, obstructive sleep apnea, Prader–Willi syndrome, sleep-disordered breathing

## Abstract

This narrative review study investigates the correlations between obesity, allergies, and sleep-disordered breathing in pediatric populations. Searches for pertinent articles were conducted on the Medline PubMed Advanced Search Builder, Scopus, and Web of Science databases from unlimited to April 2024. Sleep-disordered breathing causes repeated upper airway obstructions, leading to apneas and restless sleep. Childhood obesity, which affects around 20% of children, is often associated with sleep-disordered breathing and allergies such as asthma and allergic rhinitis. It is distinguished between diet-induced obesity (resulting from excess of diet and physical inactivity) and genetic obesity (such as is seen in Down syndrome and Prader–Willi syndrome). In children with diet-induced obesity, chronic inflammation linked to weight can worsen allergies and increase the risk and severity of asthma and rhinitis. Furthermore, the nasal congestion typical of rhinitis can contribute to upper respiratory tract obstruction and obstructive sleep apnea. A vicious circle is created between asthma and sleep-disordered breathing: uncontrolled asthma and sleep-disordered breathing can worsen each other. In children with genetic obesity, despite alterations in the immune system, fewer allergies are observed compared to the broader population. The causes of this reduced allergenicity are unclear but probably involve genetic, immunological, and environmental factors. Additional research is necessary to elucidate the underlying mechanisms. The present narrative review study emphasizes the importance of jointly evaluating and managing allergies, obesity, and obstructive sleep apnea in children considering their close interconnection.

## 1. Introduction

Obstructive sleep apnea (OSA), the most common form of sleep-disordered breathing (SDB), involves repeated obstruction of the upper airways, causing brief periods of apnea and sleep disruption. SDB in children, including snoring and OSA, can significantly affect sleep, daytime behavior, cognitive performance, physical development [1], and cardiovascular risk [2].

Polysomnography is the foremost diagnostic tool for identifying SDB [1,3]. However, alternative methods can assist in diagnosing SDB when economic resources are limited, such as sleep questionnaires (Pediatric Sleep Questionnaire) and pulse oximetry with McGill oximetry scores [4,5].

The global prevalence of OSA fluctuates, impacting approximately 1.2% to 5.7% of children. Male children are more commonly affected. In addition, conditions like obesity can increase the likelihood of developing OSA [6,7] compared to children of normal weight [8,9]. One study discovered that OSA impacted 38.7% of children with BMI z-scores below 2 in contrast to 60% of children with BMI z-scores of 2 or above [10]. 

Pediatric OSA can be classified into three phenotypes based on the underlying causes and clinical presentations. The three anatomical phenotypes in childhood OSA are adenotonsillar hypertrophy, craniofacial-based clinical phenotypes, and upper airway structure. 

(1) Adenotonsillar hypertrophy phenotype is the most common phenotype of pediatric OSA, accounting for up to 75% of cases [11]; (2) in the adult phenotype, excess weight and fat deposition in the neck and upper airway lead to high airway obstruction during sleep [12]; (3) the congenital phenotype is characterized by structural abnormalities of the skull and face and neuromuscular disorders [13]. Hypotonic neuromuscular diseases can cause hypotonia, or decreased muscle tone, in the upper airway muscles, leading to airway collapse during sleep [14]. 

Among the genetic conditions, Prader–Willi syndrome (PWS) [15] and Down syndrome (DS) [16], which also have a propensity for obesity, pose an increased risk of OSA [17]. 

Inhalant allergies can be a common comorbidity that may exacerbate SDB in children. Studies exploring the correlation between SDB and allergic conditions have amassed significant evidence elucidating the interplay between these ailments [18]. Evidence indicates a relationship between the severity of allergic rhinitis (AR) and the severity of OSA [19]. While debate persists concerning the impact of nasal inflammation on primary snoring and OSA, AR is reportedly present in 35% of children with primary snoring and 6% with OSA [19]. 

The complex relationship between obesity, genetic phenotype, and allergies in children is not explicitly investigated in the literature.

### 1.1. Diet-Induced Obesity and Sleep-Disordered Breathing

Commonly affecting 19.7% of children and adolescents, obesity is characterized by excess weight without further syndromic features [20]. Children affected by obesity exhibit a greater prevalence of OSA, with about 36% affected and 60% with the syndrome [21]. The frequency of SDB in obese children markedly surpasses that in the broader pediatric demographic, with estimations ranging from 33% to 61% compared to 1% to 3%, respectively [22].

Excess fatty tissue can lead to physical health problems in pediatric age groups, such as hypertension, orthopedic and respiratory issues, sleep disorders, as well as psychosocial risks like depression, anxiety, and low self-esteem [23]. Regular check-ups are crucial for overweight and obese children [24].

Pediatric obesity presents a notable risk factor for OSA, one which is primarily attributed to anatomical and inflammatory conditions. Fat accumulation in the neck region can potentially elevate resistance in the upper airway and disrupt respiratory function among children aged 8 to 17 years. Notably, an increase of 0.01 in the neck-to-height ratio (NHR) was correlated with a 55% escalation in obstructive AHI [25]. Obese children also exhibit anomalies during sleep, such as positive pharyngeal pressure and less pharyngeal dilatory activity [26]. 

Adenotonsillar hypertrophy is a significant risk factor for OSA in obese children, but surgical removal of the tonsils may only have a partial effect on improving the condition [27]. Some studies report a significant reduction in lung function in obese children. Obesity also increases the risk of developing obesity hypoventilation syndrome (OHS), a condition recently identified in children as well [28]. 

### 1.2. Obesity in Genetic Diseases and Sleep-Disordered Breathing

Genetic diseases associated with childhood obesity include syndromic and non-syndromic forms. Non-syndromic childhood obesity can also have genetic underpinnings, with more than 1100 independent genetic loci associated with obesity traits having been identified [29]. Syndromic obesity can be caused by genetic disorders such as PWS and DS. These syndromes are characterized by specific genetic abnormalities that contribute to obesity in affected individuals [30,31]. 

PWS and DS are both genetic conditions but exhibit significant differences. PWS and DS represent distinct genetic conditions, sharing several clinical characteristics, including obesity, muscle hypotonia, ligament laxity, and intellectual disability [32]. However, PWS is characterized by obesity due to hyperphagia and genetic abnormalities on chromosome 15, occurring in 1:10,000–1:30,000 live births [33]. Research indicates that individuals with DS exhibit a greater incidence of overweight and obesity when compared to their counterparts in the general population. Estimates suggest that 49% to 80% of young individuals with DS are overweight or obese, with this number rising to 90% by adulthood [34]. The aetiology of obesity in youth with DS is multifactorial [34].

#### 1.2.1. Obesity in Down Syndrome

DS is a genetic disorder caused by an additional copy of approximately 200 genes from chromosome 21, resulting in developmental defects that affect many physiological systems. DS is also associated with an increased risk of obesity. The prevalence of overweight and obesity collectively varies across studies, ranging from 23% to 70% [35].

Several physiological processes can contribute to this heightened risk. The increased susceptibility to obesity in children with DS is believed to stem from a combination of factors, including a diminished metabolic rate, decreased levels of physical activity, and suboptimal dietary practices [36]. An underactive thyroid gland (hypothyroidism) and a naturally slower metabolism can diminish the body’s capacity to burn calories. Additionally, consuming more calories than expended through physical activity produces a calorie surplus that can lead to weight gain [36]. Therefore, there is a significantly higher prevalence of OSA among children with DS compared to the general population. However, signs of SDB were associated with BMI z-score in the 13–15 age group (OR: 1.99 [1.06–3.72], *p* = 0.03) and 16–18 years age group (OR: 3.04 [1.22–7.59], *p* = 0.02) [37]. According to a meta-analysis, the prevalence of OSA in children with DS ranges from 69% to 76% [38]. Children with DS are at increased risk of SDB due to features commonly associated with the syndrome, such as a small oropharynx, midface hypoplasia, narrow nasopharynx, and macroglossia. Additionally, factors such as obesity, hypothyroidism, and other medical comorbidities are also known to contribute to the severity of SDB [39,40]. 

#### 1.2.2. Obesity in Prader–Willi Syndrome

PWS is a rare yet significant genetic disorder resulting from an anomaly on chromosome 15. It initially manifests with neonatal hypotonia, feeding challenges, and reduced appetite, followed by a later phase of weight gain, insatiable hunger, and uncontrollable eating tendencies, typically emerging between the ages of 2 and 3 [41]. It is considered the primary genetic contributor to obesity, affecting approximately 1 in 10,000 to 1 in 30,000 live births [33]. Children diagnosed with PWS typically demonstrate a higher ratio of body fat and display a unique distribution of fat. Individuals with PWS exhibit notably reduced lean tissue in their arms, legs, and trunks and significantly decreased trunk fat mass [42]. Patients with PWS have a particular tendency to gain weight and may develop serious health problems related to obesity, such as hypertension and OSA [30,43]. 

## 2. Purpose of the Review

This study seeks to enhance the current comprehension of the interaction between allergies and SDB in obese children. In particular, the manuscript aims to explore, through various combinations, the role of diet-induced obesity (excess of diet and physical inactivity), genetic obesity, and inhalant allergies in developing OSA, as addressed in Section 3 through Section 6 of the manuscript. In Section 3, the correlation between obesity and OSA in pediatric patients is addressed, both in those with diet-induced obesity and in those with genetic comorbidities such as DS and PWS. Section 4 explores the relationship between diet-induced obesity, allergies, and SDB in DS patients. Section 5 delves into the relationship between obesity, allergies, and SDB in pediatric patients with PWS. Finally, Section 6 addresses the potential strengths and limitations; Section 7 presents the conclusions.

Electronic searches for pertinent and updated articles were conducted on the Medline PubMed Advanced Search Builder (https://pubmed.ncbi.nlm.nih.gov/), Scopus (https://www.scopus.com/), and Web of Science (http://www.webofscience.com/) online databases (access date 26 April 2024) from unlimited to April 2024 to address these questions. The keywords used were obesity, inhalant allergies, asthma, inflammation, sleep apnea, sleep-disordered breathing, Prader–Willi syndrome, and Down syndrome.

## 3. Diet-Induced Obesity and Allergic Disease

### 3.1. Diet-Induced Obesity and Inflammation

Persistent inflammation is strongly linked with allergies and can play a pivotal role in the onset of allergic conditions and their ongoing management. Childhood obesity is increasingly being recognized as a condition with potential immediate health consequences, including the development of chronic low-grade inflammation also due to pro-inflammatory mediators released from the steatotic/inflamed liver [44]. The lower expression of IL-10 may be decisive for maintaining the low-grade inflammation status in childhood obesity [45]. 

Adipose tissue releases various compounds that influence immune function. These include multiple cytokines, such as tumour necrosis factor-alpha (TNF-α) and interleukins (IL-1, IL-6), as well as chemokines like monocyte chemoattractant protein-1 (MCP-1) and eotaxin, which specifically attract monocytes and eosinophils, respectively [46]. Furthermore, obesity is associated with increased levels of circulating acute-phase reactants, such as C-reactive protein (CRP), which further supports the presence of systemic inflammation [47]. 

Adipokines, a group of cytokines synthesized by fatty tissue, are essential for modulating metabolism and immune system functions. Among them, leptin and adiponectin exhibit contrasting functions [48]. Leptin, an adipokine secreted by fatty tissue, has been shown to have detrimental effects on OSA. The augmentation of leptin levels alongside fat mass is implicated in fostering a pro-inflammatory milieu by facilitating the Th1 immune response. Conversely, adiponectin, a hormone secreted by adipose tissue, possesses anti-inflammatory properties by inhibiting the NFκB signaling pathway. This inhibition helps protect against insulin resistance and atherosclerosis [47,49]. However, levels of adiponectin are typically reduced in obese individuals [50], which contributes to a pro-inflammatory state. This reduction in adiponectin levels can contribute to chronic low-grade inflammation associated with conditions like obesity, asthma, and AR. The interplay between these conditions and sustained low-grade inflammation is complex and multifaceted, potentially creating a cyclic pattern of inflammation and related health issues.

### 3.2. Diet-Induced Obesity and Allergies

Constant inflammation increases the risk of developing many diseases, including allergic and immunological ones. Childhood obesity is associated with various immediate and long-term health implications, including an increased susceptibility to allergies [50]. Obesity could potentially play a role in the rising occurrence of allergic conditions in children. 

In a study, participants aged 1 year and older were tested for total and allergen-specific serum IgE. Obese and overweight children were associated with higher total IgE levels. Being obese was associated with higher odds of atopy (OR = 1.26; 95% CI: 1.03–1.55). The odds ratio for sensitization to foods was particularly elevated (OR = 1.59; 95% CI: 1.28–1.98), whereas the odds for inhalant allergen sensitization were not elevated [51]. Another study revealed that individuals classified as obese (OR = 1.71; 95% CI: 1.08–2.71, *p* = 0.021) or overweight (OR = 1.62; 95% CI: 1.06–2.50, *p* = 0.026) were linked to a higher likelihood of allergic disease in prepubertal children compared to those with an average weight [52]. The correlation between childhood obesity and allergic conditions such as AR, atopic dermatitis, and chronic urticaria is somewhat contradictory yet remains noteworthy [46].

### 3.3. Diet-Induced Obesity, Allergic Rhinitis, and Obstructive Sleep Apneas

Over time, there has been a rise in the prevalence of AR. Certain studies suggest that medically diagnosed AR stood at 10.5%. In contrast, the general prevalence of self-reported current AR (within the previous 12 months) was 18.1% [53].

A direct correlation between obesity and the prevalence of AR (OR 1.33; 95% CI: 1.04–1.72; *p* < 0.05), and in particular, obesity in girls and AR (OR 1.48; 95% CI: 1.00–2.18; *p* < 0.05), has previously been reported [54]. Another study found that obese children aged 6–7-year-old had an increased prevalence of sensitization (OR = 1.84; 95% CI: 1.11–3.04; *p* < 0.05), and overweight children had an increased prevalence of a positive skin prick test (OR = 2.0; 95% CI: 1.12–3.57; *p* < 0.05). No correlation has been found between obesity and AR in 6–7-year-olds (OR = 1.05; 0.75–1.47) or 13–14-year-olds (OR = 1.56; 95% CI: 1.08–2.26) [55]. Research revealed a direct link between elevated BMI and AR, particularly among young adult females and children [46]. Obesity is recognized as a factor that can worsen AR symptoms, and simultaneously, it correlates with heightened levels of IL-1β and leptin. In particular, only parental AR (9.2-fold increase in risk), elevated leptin (11.3-fold increase in risk), and high expression of IL-1β (5.8-fold increase in risk) emerged as significant risk factors of moderate to severe persistent AR [56]. Obesity-induced increases in leptin levels may contribute to heightened inflammation and more severe symptoms through its interaction with interleukin-1 β (IL-1β) [56]. Obesity negatively influences the severity of AR when children with AR experience higher carbon monoxide (CO) and ambient particles  ≤  10 μm in diameter (PM^10^) but not in non-obese children with AR [57]. Thus, obesity among children with AR was linked to heightened susceptibility to air pollution, resulting in worsened AR symptoms [57]. The specific mechanisms that connect obesity to the deterioration of allergies and AR symptoms remain uncertain.

The link between obesity and AR, however, is not straightforward. Some studies suggest that obesity may be linked to a lower risk of AR, regardless of gender [46]. The correlation between obesity and AR in children is intricate, with contradictory results. An extensive study across the United States revealed that central obesity was associated with reduced odds of AR (OR = 0.35, 95% CI = 0.19–0.64; *p* < 0.01) in children. After stratification by sex, this association was similar in female and male children [58]. 

AR is strongly correlated with OSA development for several reasons. Firstly, nasal congestion reduces airflow through the upper airways [59], causing fatigue, snoring, and poor sleep quality. Secondly, nasal congestion can lead to mouth breathing, which causes the tongue and jaw to shift downwards. This results in a reduced pharyngeal diameter and altered position of the upper airway dilator muscles, further reducing airflow [19]. This heightened nasal resistance compels individuals to transition from breathing through the nose to breathing through the mouth, potentially contributing to upper airway collapse and apnea generation. Studies have shown that nasal obstruction, whether due to anatomical issues or chronic allergies, can destabilize the upper airways and worsen OSA by disrupting normal breathing patterns [60]. Patients with OSAS had higher nasal resistance than patients without OSAS. Persistent vasomotor and non-vasomotor rhinitis are risk factors for SDB [61]. Recent research has highlighted the higher Th17/Treg ratio in peripheral blood among children with OSA and AR than those without AR. This suggests that AR might be pivotal in fostering OSA [62].

In conclusion, the correlation between obesity, AR, and OSA syndrome underscores the intricate interconnection among these ailments. Obesity may increase vulnerability to AR and exacerbate respiratory manifestations, whereas AR could impact the pathophysiology of OSA, especially in pediatric populations.

### 3.4. Diet-Induced Obesity, Asthma, and Obstructive Sleep Apneas

**Figure 1:**
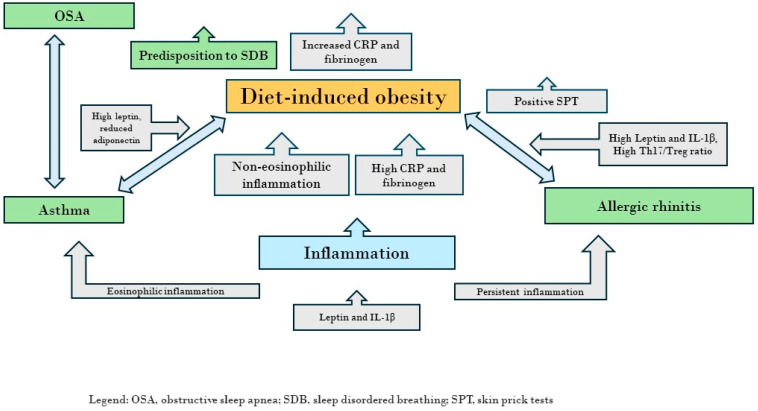
The figure shows the interconnection between allergic rhinitis, asthma, and sleep-disordered breathing in children affected by diet-induced obesity.

Figure 1 shows the interconnection between allergic rhinitis, asthma, and SDB in children affected by diet-induced obesity. 

The link between obesity and asthma in children is firmly established, with numerous epidemiological studies demonstrating a connection between the two conditions [63,64]. The co-occurrence of asthma and obesity may be due to common pathogenetic factors [65]. Being overweight was significantly associated with an increased prevalence of declared asthma in 6–7-year-olds (OR = 2.44, 95% CI: 1.52–3.91; *p* < 0.001) and 13–14-year-olds (OR = 1.66, 1.09–2.53; *p* = 0.016) [55]. Children who are persistently overweight have shown a prolonged impact on the development of incident asthma (OR = 2.47, 95% CI: 1.18–5.12) and incident AR (OR = 1.44, 95% CI: 1.12–1.84) that can persist into adolescence and early adulthood [66].

In adult patients, the interaction between obesity and asthma involves a complex interplay of inflammatory processes that can influence the onset and severity of asthma symptoms [67]. The higher adiponectin/leptin ratio in non-obese asthma patients compared to obese asthmatic subjects was the only significant difference between the two groups [68].

An affirmative correlation has been established between asthma and obesity, with inflammation as the underlying mechanism [63]. Eosinophilic inflammation was observed in asthmatic patients, while non-eosinophilic inflammation was evident in overweight patients. Notably, within the overweight children cohort, elevated levels (*p* < 0.001) of both acute inflammatory markers, CRP and fibrinogen, were detected, irrespective of asthma status [63].

Studies indicate a notable contribution of leptin to the pathophysiology of asthma among those who are obese. In a systematic review, the severity of asthma symptoms or the frequency of exacerbations tended to be greater in obese individuals, who exhibited elevated levels of leptin and reduced levels of adiponectin, in contrast to their non-obese counterparts [69]. In asthmatic children, the median serum leptin level was 9.2 ± 16.2 ng/mL. In overweight children, it was 30.6 ± 21.6 ng/mL, and in overweight asthmatic children, it was 31.1 ± 20.3 ng/mL (*p* < 0.05). However, atopy was not confirmed as an underlying mechanism of the association between asthma and overweight [70]. A meta-analysis confirmed that obesity is characterized by various alterations in both innate and adaptive immunity, with a transition from a Th2 to a Th1 immune response. This study reveals differences in immune responses mediated by T-helper cells in lean and obese children with asthma [71]. 

OSA stands as the predominant form of SDB in children, especially among those with severe asthma. A solidly established link exists between childhood asthma and OSA [72]. A review reported that several studies have highlighted a bidirectional relationship between the two conditions [65]. A concerning connection is evident between asthma and SDB in children. Uncontrolled asthma predisposes children to OSA, which can then exacerbate asthma control and increase the likelihood of asthma attacks [73]. This bidirectional relationship is further supported by the significant overlap in symptoms and underlying mechanisms (pathophysiology) between the two conditions. In patients with severe OSA, clinical evaluation for asthma should be considered [73]. 

The connection among obesity, asthma, and OSA is complex and multifaceted, potentially exacerbating each other. Obesity represents a significant risk factor for both asthma [74] and OSA. Finally, obesity and SDB often coexist, with inflammation associated with obesity potentially influencing asthma control and the reciprocal relationship between OSA and asthma or AR [58]. However, another study suggests that the independent factor associated with an increase in the risk of moderate/severe OSA was obesity (OR = 1.82, 95% CI: 1.16–2.87; *p* = 0.01), whereas current asthma was reduced (OR = 0.45, 95% CI: 0.29–0.70; *p* < 0.001) [75]. Therefore, it appears that there is a discrepancy between the reported results. While some studies indicate a clear correlation between obesity and asthma, others suggest that current asthma may not be a significant factor in increasing the risk of moderate/severe OSA in children. Further investigation may be required to fully elucidate the nature of this relationship and the factors involved.

## 4. Obesity in Down Syndrome and Allergic Diseases

### 4.1. Down Syndrome and Immunity

Patients with DS show impairments in their immune system [76,77]. In a review study, immune system disturbances in individuals with DS are characterized by continuous increases in cytokine expression, T cell activation, atypical B cell activation, and autoantibodies. Studies have indicated heightened cytokine dysregulation in DS correlates with more pronounced clinical immune dysfunction [78]. The immune dysfunction observed in DS could arise from hyperactive interferon responses, possibly due to the presence of multiple interferon receptor subunits and the autoimmune regulator (AIRE) transcription factor encoded by chromosome 21 [78,79]. 

A reduced thymus size in DS patients contributes to lymphopenia and leukopenia. Individuals with DS exhibit a notable decrease in T lymphocyte count, affecting CD4+ and CD8+ cells [78,80]. Regarding innate immunity, children with DS have a reduction in CD16+ CD56+ natural killer (NK) cells, while the number of CD16− CD57+ cells increases in both children and adults [76]. Individuals with DS also exhibit excessive expression of the SOD1 and ITGB2 genes, which are essential for neutrophil function. Nutritional deficiencies and accelerated ageing are potential causes of secondary immunodeficiency in DS [80]. 

The rate of telomere loss in patients with DS is noted to exceed that of the general population. In juveniles, the average relative telomere length in studied subjects is significantly longer than in the control group (50.46 vs. 40.56, respectively arbitrary units [AU]; *p* < 0.005). Immunogenetic abnormalities might contribute to this phenomenon by promoting increased cell division [81]. 

Additionally, an increase in CD11c*Tbet^high^ + CD21^low^B cells has been observed. These cells could potentially participate in the generation of autoantibodies. Specifically, CD11c+ B cells could contribute to autoimmunity in DS [82].

In summary, DS is associated with immune dysregulation, including steady-state increases in cytokine expression, CD4+ T cell activation, atypical B cell activation, and the presence of autoantibodies. 

### 4.2. Down Syndrome and Inflammation

Some studies suggest that trisomy 21 is characterized by an upregulation of the inflammatory response [83]. This is supported by heightened levels of pro-inflammatory (IL-2, IL-6) and anti-inflammatory cytokines (IL-10, IL-1Ra or interleuchina-1 receptor antagonist) in adults with DS [83]. Adult DS patients have a higher number of CD8+ T cells. A CD8+ T cell sub-grop of people with DS shares features observed in autoimmune and autoinflammatory conditions [84]. A study investigating prepubertal DS children evidenced brain-derived neurotrophic factor (BDNF) elevation and a marked reduction of TNF-α, TGF-β, MCP-1, IL-1α, IL-2, IL-6, IL-10, and IL-12. A different gender response was observed between males and females DS children in the analyzed cytokines [85]. Inflammatory cytokine levels have been reported to vary between adults and prepubertal children with DS. Specifically, an increase in IL-2, IL-6, and IL-10 cytokine levels has been found in adult individuals with DS [83], while a marked reduction in IL-2, IL-6, IL-10, and IL-12 has been observed in prepubertal children with DS [84]. In summary, children with DS often display a persistent pro-inflammatory condition, which likely plays a role in their heightened vulnerability to infections and inflammatory disorders [83].

### 4.3. Down Syndrome, Allergic Sensitization, and Atopic Eczema

Allergic sensitization is defined as developing specific IgE antibodies to allergens in the blood (serum) following exposure. At 19 years, those sensitized at 8 years of age or earlier had the highest risk of asthma (OR = 4.68; 95% CI: 3.15–6.97) and rhinitis (OR = 22.3; 95% CI: 13.3–37.6), and 84% had developed either asthma or rhinitis [86]. Multiple factors, including genetics, environmental triggers, and the body’s immune response, work together in a complex way to influence a person’s risk of developing allergies and becoming sensitized to allergens [87,88]. 

Patients with DS appear to have a lower allergic sensitization prevalence than non-DS children. Research indicates that allergic sensitization is much less prevalent in individuals with DS, with a prevalence of 7.6% in DS children compared to 40.2% in non-DS children [89]. This lower allergic sensitization in DS is generally reflected by diminished levels of total IgE and reduced incidence of positive skin prick tests [90]. Furthermore, the limited research on the incidence of allergic diseases in these children suggests they may be less susceptible to such conditions.

Recent studies have found a prevalence of atopic eczema in up to 5% of patients with DS, a figure comparable to that seen in the general population [91]. Nevertheless, the connection between DS, allergic sensitization, and atopic eczema is intricate and could be influenced by additional factors, including genetic predisposition and environmental exposures.

### 4.4. Down Syndrome and Wheezing

Children with DS were more than twice as likely as children in the general population group to have a moderately increased risk for asthma and respiratory allergies [92]. It has also been observed that children with DS exhibit a heightened frequency of persistent wheezing and symptoms of chronic rhinitis. In particular, the relative risk (RR) of current wheeze in DS was 2.8 (95% CI, 1.42–5.51) compared with siblings and 2.75 (95% CI, 1.28–5.88) compared with the general population [93]. Recurrent wheezing is prevalent among children with DS, likely attributed to particular factors associated with DS, but it may not be directly associated with asthma [89,93]. 

In summary, wheezing and other respiratory symptoms are common in children with DS [93,94]. The anatomical and physiological changes observed in the respiratory tract of individuals with DS probably contribute to the onset of respiratory symptoms like wheezing and coughing [92]. However, a diagnosis of asthma was found in 3.1% of children with DS, in 4.2% of siblings, and 6.7% of controls [93]. Finally, the relationship between wheezing, asthma, and DS is complex, and further research is needed to understand better the underlying factors contributing to respiratory symptoms in this population.

### 4.5. Down Syndrome, Allergies, Asthma, and Obstructive Sleep Apneas

**Figure 2:**
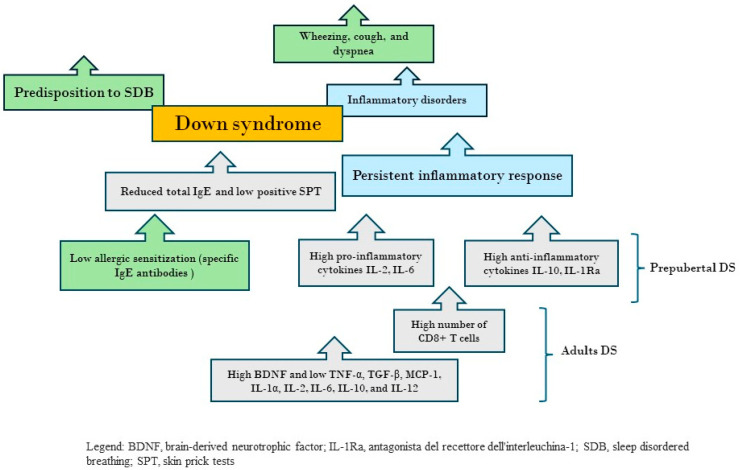
The figure shows the interconnection between allergies, respiratory symptoms, and sleep-disordered breathing in children with Down syndrome.

Figure 2 shows the interconnection between allergies, respiratory symptoms, and SDB in children with DS. 

The specific relationship between allergies and OSA in children with DS has been less directly investigated. Existing literature suggests that the immune system in individuals with DS may function differently [78,89,95], potentially affecting the low prevalence and severity of allergies [89]. However, in children with DS, an elevated prevalence of OSA (53%) [96] and SDB were frequently observed (63%) [96]. For this reason, early screening for OSA in children with DS is vital [97]. However, a large multicenter cohort study confirmed that allergic sensitization is much less prevalent in DS (7.6%) than in non-DS (40.2%) children. In contrast, symptoms such as wheezing, cough, and dyspnea are prevalent in DS [89]. For this reason, other causes besides allergies should be considered in the case of respiratory symptoms in children with DS. Finally, the correlations between DS, allergies, asthma, and OSAS are intricate, and additional research is warranted to gain a deeper understanding of the underlying factors contributing to respiratory symptoms in individuals with DS.

## 5. Obesity in Prader–Willi Syndrome and Allergic Diseases

### 5.1. Prader–Willi Syndrome and Immunity

Research suggests that individuals with PWS may have an altered immune profile. Individuals with PWS have an overactivation of the innate immune system, independent of central adiposity and insulin resistance. PWS subjects showed significantly higher IL-6 (*p* < 0.01). Neutrophil activation markers CD66b and CD11b were higher in PWS compared to obese subjects (*p* < 0.01), reflecting an activated innate immune system [98]. Furthermore, a study found that individuals with PWS exhibited increased levels of matrix metalloproteinase (MMP-9; *p* < 0.001) and myeloperoxidase (MPO; *p* < 0.001) alongside reduced levels of macrophage inhibitory factor (MIF; *p* < 0.001) [99]. These immune dysregulations in PWS may contribute to the increased susceptibility to inflammation-related diseases observed in this population. 

### 5.2. Prader–Willi Syndrome and Inflammation

Four chemokines (MCP1, MDC, Eotaxin, RANTES) showed notably elevated levels in children with PWS compared to the control group. These chemokines are known to be pro-inflammatory. Interestingly, no correlation was observed between chemokine levels and total body fat percentage [100]. Increased concentrations of IL-1β and IL-13 were detected in patients with PWS and are associated with various psychopathological symptoms. Additionally, most serum inflammatory cytokines, including IL-1β, IL-2R, IL-12p70, and TNF-α, are increased in PWS. Notably, there is a significant elevation in CD16+ monocytes, a type of immune cell, among individuals diagnosed with PWS [101]. These findings suggest that activation of the innate immune system, the body’s first line of defence, maybe a specific feature of PWS [98,99].

### 5.3. Prader–Willi Syndrome and Allergic Diseases

**Figure 3:**
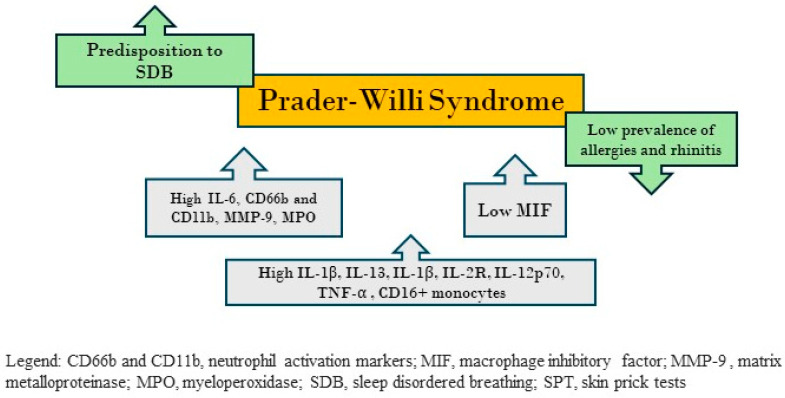
The figure shows the interconnection between allergies and sleep-disordered breathing in children with Prader–Willi syndrome.

Figure 3 shows the interconnection between allergies and SDB in children with PWS.

Individuals with PWS may have a lower prevalence of allergies and rhinitis than the general population despite exhibiting modified ventilatory control and various other factors that predispose them to SDB. Although obesity is prevalent in PWS, the relationship between obesity and pulmonary function in children is intricate [102]. In addition, food allergies and atopy may be less common in PWS than in the general population [103].

Finally, the relationship between PWS and allergic diseases is not well-studied, and further research is needed to understand the potential impact of PWS on the immune system and the development of allergic diseases.

### 5.4. Comparison Analysis of the Reviewed Studies

The studies we have thoroughly examined explore the intricate connections between childhood obesity and OSA combined with allergies, asthma, and AR. 

Frist, regarding diet-induced obesity, inflammation, and allergies [50], the studies underscore the significant link between persistent inflammation [45] and conditions like obesity and allergies [44]. They emphasize how pro-inflammatory mediators released from inflamed liver and adipose tissue play a crucial role [44,46,48,50]. However, it is worth noting that relying solely on published literature might introduce bias, potentially neglecting other genetic syndromes that affect obesity and allergies. Additionally, the interactions among obesity, allergies, and respiratory issues are multifaceted and pose challenges due to various confounding factors.

Second, concerning obesity, allergies, and OSA, the studies highlight how childhood obesity heightens susceptibility to allergies [51], evidenced by a higher prevalence of allergic sensitization and atopy among obese children. Despite this, the correlation between obesity and AR appears complex, with conflicting findings across different studies [51,52]. Moreover, while obesity worsens AR symptoms [46,56], the precise mechanisms linking obesity, AR symptoms, and OSA remain debated [19,46,60,61].

Moving on to obesity, asthma, and OSA, epidemiological studies firmly establish a link between obesity and asthma in children [63,64], with obesity contributing to a persistent low-level inflammatory state [63]. OSA is prevalent among children with asthma, suggesting a bidirectional relationship between these two conditions [58,74]. The intricate interplay among obesity, asthma, and OSA exacerbates respiratory symptoms, highlighting the need for further research to understand underlying factors better.

In terms of DS and allergic diseases, individuals with DS exhibit immune dysregulation characterized by cytokine dysregulation and alterations in innate immunity [76,78,80]. Although allergic sensitization seems less prevalent in DS [89], the relationship between DS, allergic diseases, and respiratory symptoms is complex and influenced by various factors [89,92,93]. Finally, a specific relationship between allergies and OSA in children with DS requires further research. 

Finally, for PWS and allergic diseases, individuals with PWS display altered immune profiles, including overactivation of the innate immune system and elevated levels of inflammatory cytokines [100,101]. Despite the prevalence of obesity in PWS, the relationship between obesity and allergic diseases is understudied, necessitating further research.

## 6. Strengths and Limitations

This review provides a detailed examination of how obesity affects health, especially in conditions like DS and PWS, and delves into its connection to allergies and sleep issues. It consolidates information from studies and data sources to offer a broader understanding. Healthcare professionals and researchers can utilize this resource to gain insights into how obesity, allergies, and breathing problems intersect, particularly in children.

However, it primarily discusses DS and PWS, potentially overlooking other genetic conditions that also contribute to obesity and allergies. As it relies mainly on published research, there may be a bias towards studies with positive results, disregarding those with less significant findings. Additionally, since it focuses on children with DS and PWS, its applicability to other groups may be limited. Finally, the relationships between obesity, allergies, and breathing issues are intricate and involve numerous factors, making them challenging to comprehend fully. 

## 7. Conclusions

This study highlights several key points regarding the interconnection between obesity, allergic diseases, and SDB. Diet-induced obesity is associated with the onset of persistent low-grade inflammation and a higher prevalence and severity of allergies like asthma and AR. Various inflammatory pathways and adipokines mediate this relationship. Additionally, the severity of AR is correlated with diet-induced obesity, and nasal congestion in AR may contribute to upper airway obstruction and the development of SDB. In diet-induced obesity, there is a bidirectional relationship between asthma and SDB, with uncontrolled asthma increasing the risk of SDB and vice versa.

In cases of genetic obesity such as DS and PWS, despite alterations in the immune system, allergies are less common compared to the general population. While the precise causes behind this phenomenon in PWS and DS remain unclear, a combination of genetic, immunological, and environmental factors is likely involved. 

Our study contributes to the body of scientific literature on obesity and its comorbidities, including OSA and allergies, laying a foundation for further research. By investigating the role of allergies in genetically determined obesity conditions, we identify a gap in current research, suggesting an area of interest for future studies. While the correlation between dietary obesity, OSA, and allergies has been adequately addressed in the literature, the role of allergies in genetically determined obesity conditions remains insufficiently explored. Long-term observational studies tracking individuals from childhood to adulthood could offer valuable insights into the progression of AR, asthma, and OSA concerning obesity. Clinical trials assessing the effectiveness of weight management interventions in alleviating respiratory symptoms and enhancing outcomes in individuals with obesity and comorbid respiratory conditions are warranted.

Finally, these findings emphasize the significance of adopting a comprehensive approach to evaluating and addressing allergic conditions and SDB in patients with either diet-induced or genetic obesity, considering the complex interconnections between them.

## Data Availability

Not applicable.
